# Failure Patterns of Recurrence and Metastasis After Intensity-Modulated Radiotherapy in Patients With Nasopharyngeal Carcinoma: Results of a Multicentric Clinical Study

**DOI:** 10.3389/fonc.2021.693199

**Published:** 2022-02-11

**Authors:** Sixia Chen, Dong Yang, Xueyin Liao, Ying Lu, Bin Yu, Meng Xu, Ying Bin, Pingting Zhou, Zhendong Yang, Kang Liu, Rensheng Wang, Tingting Zhao, Min Kang

**Affiliations:** ^1^ Department of Radiation Oncology, The First Affiliated Hospital of Guangxi Medical University, Nanning, China; ^2^ Guangxi Tumor Radiation Therapy Clinical Medical Research Center, Nanning, China; ^3^ Department of Radiation Oncology, The Fourth Affiliated Hospital of Guangxi Medical University, Liuzhou, China; ^4^ Department of Radiation Oncology, The People’s Hospital of Liuzhou, Liuzhou, China; ^5^ School of General Practice, Guangxi Medical University, Nanning, China

**Keywords:** intensity-modulated radiotherapy (IMRT), nasopharyngeal carcinoma (NPC), distant metastasis, recurrence, failure pattern

## Abstract

**Purpose:**

This study aimed to explore factors associated with recurrence and metastasis after intensity-modulated radiotherapy (IMRT) in patients with nasopharyngeal carcinoma (NPC) and provide evidence for NPC treatment.

**Methods:**

We retrospectively analysed the treatment dose and survival outcomes of 645 patients with nasopharyngeal carcinoma without distant metastases treated with IMRT for the first time at three treatment centres in the Guangxi Zhuang Autonomous Region, China, between January 2009 and December 2012.

**Results:**

There were 9.3% of patients (60/645) had recurrence and 17.5% (113/645) had distant metastasis 5 years after treatment. The 1-year, 3-year and 5-year local recurrence rates were 0.9%, 6.5% and 9.0% respectively. And the 1-year, 3-year and 5-year distant metastasis rates were 3.4%, 10% and 17.2%, respectively. In the 60 patients with recurrence, the in-field, marginal-field, and out-field recurrence rates were 93.3% (56/60), 5.0% (3/60) and 1.7% (1/60), respectively. Recurrence failures occurring within the first three years after treatment accounted for 81.7% (49/60). In the 113 patients with metastasis, the size of the cervical lymph node, the presence of lower cervical lymph node metastasis, the residual cervical lymph node size and the time of residual cervical lymph node complete response (CR) were independent prognostic factors for DMFS (*P <*0.05).

**Conclusion:**

Most recurrences occured in the first three years after IMRT. In-field recurrence was the most common pattern for loco-regional failure of NPC treatment. The risk of distant metastasis was positively correlated with higher N stage, lower neck nodal metastasis, larger size of cervical lymph nodes, and longer time to response for residual NPC in cervical adenopathy.

## Introduction

Nasopharyngeal carcinoma (NPC) is a common malignant tumor, with higher incidence in southeastern China than that in other regions of China. In recent years, intensity-modulated radiotherapy (IMRT) has gradually replaced traditional two-dimensional radiotherapy (2D-RT) for nasopharyngeal carcinoma (1).

Comprehensive treatment with IMRT as the primary treatment approach significantly improves the prognosis of patients with a local control rate over 90%, and overall survival rate over 80% (2–6). Although radiotherapy alone has excellent performance in the treatment of early nasopharyngeal carcinoma, radiotherapy is a local treatment and has limited effect on distant metastasis. Chemotherapy can effectively kill distant metastatic subclinical cancer cells. Theoretically, radiotherapy combined with chemotherapy can reduce the distant metastasis rate and improve the long-term survival rate of nasopharyngeal carcinoma. According to the 2021 National Comprehensive Cancer Network (NCCN) guidelines for nasopharyngeal carcinoma,concurrent chemoradiotherapy (CCRT) is the standard treatment for patients that are stage II-IVb and Cisplatin (CDDP)-based chemotherapy is the regimen most commonly used in recent years.

However, distant metastasis and recurrence remain the primary reasons for failure (7–10). The clinical characteristics of recurrent and metastatic NPC are multi-specific (7–10). Therefore, a deeper understanding of the clinical feature characteristics of recurrent and metastatic NPC is crucial,which can be helpful in the early diagnosis and treatment of NPC. In this multicenter study, we reviewed a large cohort of patients to explore the factors related to NPC recurrence and distant metastasis after IMRT, which could provide evidence for NPC treatment.

## Material and Methods

### Patients

We retrospectively analyzed 645 patients with untreated, non-distant metastatic, newly histological confirmed NPC treated with IMRT from January 2009 to December 2012 at three hospitals (the First Affiliated Hospital of Guangxi Medical University, 424; the Fourth Affiliated Hospital of Guangxi Medical University, 167; and the People’s Hospital of Liuzhou, 54). All patients underwent a detailed clinical and laboratory examinations, pre- and post-treatment magnetic resonance imaging (MRI) of the head and neck region, nasopharyngoscopic biopsy, chest X-ray or CT, abdominal ultrasound or CT, and bone scan for exclusion of distant metastases. The study was approved by the ethics review board of Guangxi Medical University.

### Clinical Staging

All MRI images were reviewed independently by two radiologists specialized in head and neck MRI., Any disagreements were resolved by consensus.and by referencing relevant patient clinical information (such as cranial nerve palsy, lymph node size, etc.). Tumors were staged according to the seventh edition UICC/AJCC Staging System (11). The location of lymph node was identified according to the RTOG nodal classification criteria (2013 edition) (12). If MRI showed lymph node metastasis, but palpation was not found, then the MRI prevailed.

### Diagnostic Criteria for Distant Metastasis and Local and Regional Recurrences After Radiotherapy

Diagnosis of metastasis to the lung, liver, brain or chest was primarily determined with imaging result, and then subjected to a pathological confirmation. Our diagnostic approach included analysis of tumor markers and radiography methods (such as CT, PET or MRI). Bone metastasis was also confirmed through two radiography methods and excluding the metastasis from another primary tumor.

Local recurrence was defined as the appearance of a new pathologic biopsy-confirmed tumor at six months after radiotherapy. For cases in which pathological evidence could not be acquired, local recurrence could be identified according to the MRI examinations and the clinical evaluation. Lymph node residual was defined as the existence of the lymph node at the end of radiotherapy. Regional recurrence was defined as the appearance of the positive lymph node verified by fine needle aspiration biopsy (FNA), excision, or clinical and multi-imaging examination at six months after radiotherapy (13).

Tumor volume with recurrence (Vr) in 60 NPC patients was assessed by two experienced radiation doctors. The Vrs were normalized to the initial treatment plan to compare the V95 (volume of 95% isodose lines) of the Vr and the initial treatment plan, which was divided into: (1) In-field recurrence: ≥95% Vr within the 95% isodose lines of the primary target region. (2) Marginal-field recurrence: 20%≤Vr<95% within the 95% isodose lines of the primary target region. (3) Out-field recurrence: <20% Vr within the 95% isodose lines of the primary target region.

### Treatment

After identification of the gross tumor volume(GTV)by MRI, contrast-enhanced CT was performed for target delineation and treatment planning (11, 14). Based on the varying conditions of each clinical tumor center, prescription doses were delivered according to planning target volumes (PTV), which were determined by adding a 3- to 5-mm margin to each target region: 69.96–74.58 Gy to the GTV in the nasopharynx and positive neck nodes; 62.0–66.03 Gy to the high-risk clinical target volume (CTV1); 51.15–56.1 Gy to the low-risk clinical target volume and lymphatic drainage region of the neck (CTV2). All targets were treated once daily, 5 times a week, for a total fraction of 30-33. The dose constraints of organ at risk (OAR) and plan evaluation were determined according to the Radiation Therapy Oncology Group (RTOG) 0615 and the RTOG 0225 protocols (15).

Of the 645 cases, 12.7% (82/645) received radiotherapy (RT), 28.1% (181/645) received concurrent chemoradiotherapy (CCRT), 53.2% (343/645) received CCRT-neoadjuvant chemotherapy (NACT) and/or adjuvant chemotherapy (ACT), and 6.0% (39/645) received RT + NACT and/or ACT. CCRT was carried out using platinum-based chemotherapy (cisplatinum, nedaplatin, and carboplatin); NACT and ACT were performed using platinum-based combined chemotherapy (paclitaxel/platinum, paclitaxel/fluorouracil/platinum, and fluorouracil/platinum).

### Follow-Up

Follow-up evaluations occurred every 3 months during the first 3 years and every 6 months thereafter. The follow-up time was calculated from the date of treatment completion to the date of the last contact or death. By the follow-up day of December 31th 2017, the median follow-up time was 62 months (with a range of 11-95 months) with a follow-up rate of 96.6%. Physical examination, chest X-ray, abdomen ultrasound, fiber nasopharyngoscopy, and laboratory analysis were performed at each follow-up. MRI of the head and neck was performed every 6 months. For patients with suspicious distant metastasis on physical examination, CT scan of the chest and abdomen and bone scintigraphy were performed to confirm the metastasis. And the patients will undergo positron emission tomography/computed tomography (PET/CT) examinations if necessary.

### Statistical Analysis

Statistical analyses were conducted using SPSS 19.0 software. The Kaplan-Meier method was used to calculate survival rate, and the log-rank test was used to compare survival outcomes. The Cox model was used for multivariate prognostic analysis. Host factors (such as the WHO classification, age, gender, chemotherapy treatment, and TNM staging) were set as covariates in the multivariate analysis. The main analysis indicators included overall survival (OS) rate, regional recurrence-free survival (RRFS), local relapse-free survival (LRFS), and distant metastasis-free survival (DMFS). *P* values < 0.05 were considered statistically significant.

## Results

### Patient Demographics

**Figure 1:**
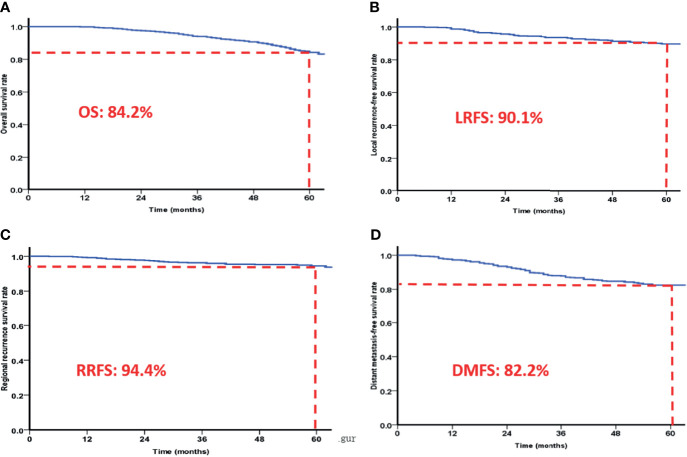
The 5-year overall survival rates **(A)**, local recurrence-free survival rates **(B)**, regional recurrence-free survival rates **(C)** and distant metastasis-free survival rates **(D)** of patients based on IMRT.

A total of 645 NPC patients were included in this study, with median age of 46 years (range: 18-78 years). There were 478 male patients and 167 female patients (male/female ratio of 2.86/1). According to the seventh edition of the UICC/AJCC Staging System, 2.5% (16/645), 13.0% (84/645), 47.8% (308/645), 29.8% (192/645), and 7.0% (45/645) of the patients were classified as stage I, II, III, IVa, and IVb, respectively. Patients with stage T1, T2, T3, and T4 NPC accounted for 6.4% (41/645), 20.2% (130/645), 40.6% (262/645), and 32.9% (212/645), respectively. Patients with stage N0, N1, N2, and N3 accounted for 13.5% (87/645), 34.7% (224/645), 44.8% (289/645), and 7.0% (45/645), respectively. The 5-year OS, RRFS, LRFS, and DMFS rates were 84.2%, 94.4%, 90.1%, and 82.8% **(** **Figure 1** **)**, respectively. After radiotherapy treatment, 58.0% (374/645) of patients achieved complete remission and 42.0% (271/645) of patients achieved partial remission. Of the patients with partial remission, 74.2% (201/271) had detectable primary tumor residue, 47.2% (128/271) had cervical lymphatic lesion residue, and 21.4% (58/271) had both primary tumor residue and cervical lymphatic lesion residue.

### Clinical Treatment Outcome

Of the 645 NPC patients treated with IMRT, recurrence occurred in 60 patients. Of the patients with recurrent NPC (rNPC) the rates of local recurrence (except for retropharyngeal lymph nodes), regional node recurrence, local and regional node recurrence, and retropharyngeal lymph node recurrence, were 55.0% (33/60), 33.3% (20/60), 6.7% (4/60), and 5.0% (3/60), respectively. 81.7% (49/60) of local failures occurred within the first 3 years after treatment completion. The time from completion of the treatment to the first recurrence ranged from 9 to 75 months, with a median of 27 months. The patients with a recurrence time of less than 6 months, 6 to 12 months, 12 to 18 months, 18 to 24 months, 24 to 36 months, 36 to 60 months, and more than 60 months was 0% (0/645), 0.9% (6/645), 2.3% (15/645), 1.6% (10/645), 2.8% (18/645), 1.4% (9/645), and 0.3% (2/645), respectively. The most common site for recurrence was the nasopharynx, local anatomical sites are the pterygomaxillary fossa, cavernous sinus, parapharyngeal space, ruptured hole, etc. According to the diagnostic criteria for local and neck node regional recurrences, of the 60 patients with local recurrent NPC, 93.3% (56/60) were classified as a GTV in-field recurrence with a radiotherapy dose of 70-74 Gy, and 5.0% (3/60) were classified as a GTV marginal field recurrence with a radiotherapy dose of 54-66 Gy. Only 1 patient was classified as a GTV out-field recurrence. Among the patients that had recurrence, 41 were male (68.3%) and 19 were female (31.7%), giving a male/female ratio of 2.158. The mean age of these patients was 46 years old (with a range of 18-78 years old). The 1-year, 3-year, and 5-year locoregional recurrence rates were 0.9%, 6.5%, and 9.0%, respectively. The 1-year, 3-year, and 5-year locoregional control rates were 99.1%, 93.4%, and 90.1%, respectively.

Of the 645 patients with NPC undergoing intensive radiotherapy, 113 patients had distant metastases within 5 years of radiotherapy. The median age of patients with distant metastases was 47 years (range: 20-74 years). The median time of distant metastasis occurrence was 24 months (range: 6-79 months) after radiotherapy. Among the 113 patients with distant metastases, there were 68 patients with bone metastases (including 25 cases of bone metastasis alone, 16 cases of bone and liver metastasis, 7 cases of bone and lung metastasis, and 9 cases of bone and brain metastasis), 7 cases of brain metastasis, 24 cases of liver metastasis, 12 cases of lung metastasis, 2 cases of axillary lymph node metastasis, and 11 cases of metastases to multiple organs. There were 82 males (72.6%) and 31 females (27.4%) who had distant metastases, giving a male to female ratio of about 2.65/1.

By the last follow-up day (December 31, 2017), 112 patients in the sample had died. Seventy-eight patients died of distant metastasis, 16 died of recurrence, 7 died of recurrence and metastasis, and 10 died of other causes (including 1 case of car accident, 3 cases of cerebrovascular diseases, 2 cases of cardiovascular disease, 1 case of pulmonary infection, and 1 case with an unknown cause of death).

### Univariate Analysis of the Effect of General Clinical Factors on DMFS and RRFS

Univariate analysis was conducted to determine the effect of age, sex, pathological tumor type, chemotherapy (yes/no), radiotherapy dose, and short-term efficacy of treatment on DMFS and RRFS. The results showed that lymph node residue at the end of radiotherapy significantly impacted 5-year DMFS (*P <*0.05). However, the primary tumor residue and lymph node residue after radiotherapy did not significantly impact 5-year RRFS (*P >*0.05). The variables of age, sex, pathological type, chemotherapy, primary tumor, and lymphatic lesion radiotherapy dose had no significant effect on 5-year DMFS or RRFS (*P*> 0.05). In addition, primary tumor residue after radiotherapy did not impact 5-year DMFS (*P*> 0.05, **Table 1**).

**Table 1:** Univariate analysis of general clinical factors on DMFS and RRFS.

Factor	Number	Five-year DMFS (%)	X^2^	P value	Five-year RRFS (%)	X^2^	P value
Age	–	–	0.568	0.451	–	2.202	0.138
<46	315	83.9	–	–	94.7	–	–
≥46	330	81.7	–	–	91.7	–	–
sex	–	–	0.170	0.680	–	1.15	0.284
Male	478	82.8	–	–	91.4	–	–
Female	167	81.4	–	–	88.6	–	–
Pathological types	–	–	0.061	0.805		0.000	0.999
WHO I III	54	86.6	–	–	93.2	–	–
WHO II	591	82.4	–	–	93.2	–	–
Chemotherapy			0.349	0.555		2.323	0.127
No	132	81.0	–	–	98.2	–	–
Yes	513	83.3	–	–	92.7	–	–
Dose for primary lesion target(cGy)	–	–	2.523	0.112	–	1.054	0.305
<7125	315	83.5	–	–	92.1	–	–
≥ 7125	330	87.8	–	–	94.4	–	–
Dose for lymph node target(cGy)	–	–	2.295	0.130	–	0.009	0.923
<6820.5	326	87.7	–	–	93.1	–	–
≥ 6820.5	319	83.6	–	–	93.4	–	–
Residue at the end of radiotherapy	–	–	–	–	–	–	–
T residue	211	80.8	3.120	0.084	89.0	2.870	0.105
N residue	118	78.9	7.298	0.007*	88.9	2.013	0.114

DMFS, distant metastasis-free survival; RRFS, regional recurrence-free survival. *Statistically significant.

### Relationship of Recurrence Rates and Metastasis Rates With Clinical Stages

Of the 645 patients, the local recurrence rates of T stage 1, 2, 3, and 4 were 4.9% (2/41), 9.2% (12/130), 8.0% (21/262), and 11.8% (25/212), respectively. The regional recurrence rates of N stage 0, 1, 2, and 3 were 16.1% (14/87), 8.0% (18/224), 7.6% (22/289), and 13.3% (6/45), respectively. The recurrence rates of clinical stage I, II, III, and IV were 0% (0/6), 13.1% (11/84), 7.1% (22/309), and 11.4% (27/236), respectively. The 5-year distant metastasis rates of T stage 1, 2, 3, and 4 were 7.3% (3/41), 16.2% (21/130), 15.3% (40/262), and 21.7% (46/212), respectively. N stage 0, 1, 2, and 3 were 6.9% (6/87), 12.5% (28/224), 21.1% (61/289), and 35.6% (16/45), respectively. The distant metastasis rates of clinical stage I, II, III, and IV were 6.3% (1/16), 13.1% (11/84), 15.2% (47/309), and 22.5% (53/236), respectively.

Comparison of local and regional recurrence rates of different clinical stages, indicated that there were no significant differences in the local and regional recurrence rates among T, N, and clinical stages (**Table 2**). Comparing the distant metastasis rates of different clinical stages showed that N stage was an independent prognostic factor for 5-year DMFS (*P <*0.05).

**Table 2:** Univariate analysis of staging on DMFS and RRFS.

Factor	Number	Five-year DMFS (%)	X^2^	P value	Five-year RRFS (%)	X^2^	P value
Clinical stage	–	–	1.710	0.635	–	8.829	0.066
I	16	93.8	–	–	95.5	–	–
II	84	87.2	–	–	93.3	–	–
III	309	84.9	–	–	91.9	–	–
IV	236	77.5	–	–	85.9	–	–
T stage	–	–	0.587	0.899	–	3.631	0.304
T1	41	92.6	–	–	96.8	–	–
T2	130	83.5	–	–	96.6	–	–
T3	262	84.6	–	–	89.5	–	–
T4	212	78.0	–	–	85.3	–	–
N stage	–	–	16.517	0.001*	–	0.515	0.916
N0	87	93.1	–	–	88.9	–	–
N1	224	87.4	–	–	86.5	–	–
N2	289	78.8	–	–	84.6	–	–
N3	45	64.7	–	–	85.8	–	–

DMFS, distant metastasis-free survival; RRFS, regional recurrence-free survival. *Statistically significant.

### Univariate Analysis of the Effect of N Staging and Cervical Lymphatic Residue on Distant Metastasis

We conducted univariate analysis of N-staging factors for 558 patients with cervical lymph node metastasis using DMFS as the observation index. Lymphatic metastasis size and lower cervical lymph node metastasis had a significant effect on 5-year DMFS (*P <*0.05). In contrast, one or two lateral cervical lymph node metastases, extracapsular spread, necrosis, or skip metastasis did not significantly affect 5-year DMFS (**Table 3**).

**Table 3:** Univariate analysis of N staging factors on DMFS.

Factor	Number	Five-year DMFS (%)	X^2^	P value
Unilateral	235	86.7	1.698	0.183
Bilateral	323	82.8	–	–
Lymph node size	–	–	21.532	0.000*
≤3cm	322	83.9	–	–
3cm-6cm	208	77.0	–	–
≥6cm	28	54.7	–	–
Necrosis	–	–	1.669	0.196
Yes	383	78.5	–	–
No	175	83.7	–	–
Skip metastasis	–	–	2.279	0.131
No	544	82.5	–	–
Yes	14	66.7	–	–
Lymph node Region/Area	–	–	24.347	0.000*
Upper cervical	519	82.2	–	–
Lower cervical IVa/IVb/Vb/Vc	39	55.5	–	–
Extracapsular spread	–	–	0.009	0.925
No	312	82.1	–	–
Yes	246	82.3	–	–

*Statistically significant.

In all 558 patients with cervical lymph node metastasis, 22.90% (128/558) had residual cervical lymphatic nodes after radiotherapy. In 82.8% (106/128) of the patients, the lymph node residue was in region II. Univariate analysis showed that residual lymphatic node size and complete response time of the residual lymphatic nodes had effects on 5-year DMFS (P<0.05), while the location of the residual lymphatic nodes, the number of residual lymphatic nodes, and unilateral or bilateral distribution had no effect on 5-year DMFS (**Table 4**).

**Table 4:** Univariate analysis of 128 cases of cervical lymph node residual lesion on DMFS.

Factor	Number	Five-year DMFS (%)	X^2^	P value
Unilateral or bilateral	–	–	0.439	0.507
Unilateral	89	86.0	–	–
Bilateral	39	81.5	–	–
Number of residual lymph node	–	–	0.359	0.459
<3	108	89.7	–	–
≥3	20	83.7	–	–
Size of residual lymph node (cm)	–	–	13.952	0.000*
≤1	95	91.1	–	–
>1	33	65.9	–	–
Region/area of residual lesion	–	–	0.339	0.560
Ib	2	100	0.339	0.560
II	104	86.1	1.347	0.246
III	10	80.0	0.172	0.678
VIIa(RP)	21	89.5	0.420	0.517
Complete response time(months)	–	–	9.765	0.008*
≤3	96	90.3	–	–
>3-≤6	20	68.2	–	–
>6	12	66.7	–	–

*Statistically significant.

### Multivariate Analysis

**Figure 2:**
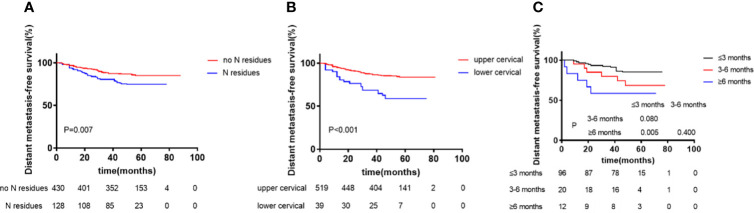
Kaplan–Meier curves of distant metastasis-free survival according to N residues at the end of radiotherapy **(A)**, Region of Lymph node (upper vs lower cervical) **(B)**, and Complete response time of residual lymphatic node (≤3 months vs 3-6 months vs ≥6 months) **(C)**. p values were calculated with the log-rank test.

We analyzed the factors found to have significant (*P <*0.05) effects in univariate analysis using the Cox proportional hazard model. Patients’ age, sex, chemotherapy treatment, WHO classification, and clinical stage were used as covariates in the regression and 5-year DMFS and RRFS were designated as the observation index. Multivariate analysis showed that N staging and cervical lymphatic residue after radiotherapy were independent prognostic factors of 5-year DMFS (*P <*0.05). Lymphatic metastasis in the lower neck IVa/IVb/Vb/Vc areas in the N stage and the size of the cervical lymphatic nodes were independent prognostic factors of 5-year DMFS (*P <*0.05). The size of cervical lymphatic residues and the complete response time for residual lymphatic lesions were independent prognostic factors of 5-year DMFS (*P <*0.05, **Table 5** and **Figure 2**).

**Table 5:** Multivariate analysis: Cox proportional hazard model on 5-year DMFS.

Factor	5-year DMFS	
	P value	HR(95% CI)
N staging (N0-1 vs N2-3)	0.000*	1.920 (1.543-2.389)
N residues at the end of radiotherapy	0.027*	1.648 (1.059-2.565)
Lymph node size(≤3cm vs 3-6cm vs ≥6cm)	0.002*	1.680 (1.203-2.374)
Region of Lymph node (upper vs lower cervical)	0.016*	1.994 (1.135-3.501)
Size of residual lymphatic at the end of radiotherapy(>1cm vs ≤1cm)	0.005*	3.803 (1.501-9.636)
Complete response time of residual lymphatic node (≤3 months vs 3-6 months vs ≥6 months)	0.001*	2.952 (1.583-5.503)

*Statistically significant.

## Discussion

Intensity modulated radiotherapy (IMRT) has gradually replaced 2D or 3D conformal radiotherapy (CRT) as the mainstream treatment modality. and dramatically improved the prognosis of NPC. The 5-year LRFS, DMFS, DFS, DSS, and OS rates for NPC treated with IMRT were 92.3%, 83.6%, 76.4%, 85.1%, and 83.7%, respectively, as previously reported (16). In the present study involving 645 NPC patients treated with IMRT, the 5-year OS, RRFS, LRFS, and DMFS rates were 84.2%, 94.4%, 90.1%, and 82.8%, respectively, which is consistent with other reports. Compared to conventional radiotherapy and 3D-CRT, IMRT achieves satisfactory target coverage, protection of normal tissues, higher overall survival rate, and better local control rate in NPC patients. However, local/regional recurrence and distant metastasis remain the main causes of failed for NPC (7, 17–19).

Our study indicated that the recurrence rate was 9.3% (60/645), with a mean recurrence age of 46 years (18-78 years),which was consistent with the other reports (20, 21). The recurrent constituent ratio declined gradually from the completion of the treatment. Our study revealed that 81.7% (49/60) of local failures occur within the first 3 years after treatment. Based on the analysis of 337 NPC patients that had recurrence from 1999 to 2004, the calculated recurrence rates at 2 years and 5 years were 48.7% and 83.1%, respectively (22). Thus, follow-up after treatment in the first 5 years is essential for NPC patients. We recommend a follow-up every 3 months for the first 3 years after treatment, every 6 months from the third to fifth year, and annually thereafter. Age and gender should be considered for the distant metastasis of NPC patients after IMRT (23, 24), which showed that age was not associated with distant metastasis, but males were 3.7 times more likely to develop distant metastasis than females. Of the 113 patients with distant metastasis, the ratio of male to female was 2.65/1. In some reports (25, 26), more than 70% of distant metastasis occurred within three years after IMRT, and tend to be stable after 3 years. Of the 645 patients in our current study, 17.5% developed distant metastasis, 73.4% of which occurred within 3 years.

Our results indicated that there were no significant differences in local and regional recurrence rates among T, N, and clinical stages, which is consistent with previous reports. Wu L. et al. (27). concluded that there was no significant difference between T stage in NPC local recurrence after IMRT. Data from Li L.’s study (28) also indicated that T stage is not an independent prognostic factor for NPC recurrence. The primary causes of tumor recurrence may be due to the biological properties of cancer cells. Tumor may result in a leaky vascular supply and cell hypoxia, which causes an insensitivity to radiation or radio-resistance of tumor cells (29, 30). Additionally, a large tumor volume also results in a smaller demarcation, limiting the enhancement of the radiotherapy dose. Several methods of promoting control rate could be used, such as exploring areas of insensitivity to radiation or radio-resistance through the use of biological imaging methods such as PET/CT (31), using radiosensitizers, and enhancing the local radiotherapy dose (32, 33). Wu T (34) noted that the total dose of re-irradiation is an independent prognostic factor for the survival of recurrent NPC patients. However, in a study by Wu L (27), for these clonogenic cells that are insensitive to radiation, blindly increasing the radiotherapy dose may result in an excess of radiotherapy without reducing the local recurrence, which increases long-term radiation injury and decreases quality of life.

N staging is related to poor prognosis (35). However, reports vary concerning how specific factors affect NPC prognosis. Yi et al. (36) reports that Ib lymph node enlargement of the carotid sheath area due to tumor invasion is an independent factor that affects prognosis. Tang et al. (37) reports that posterior pharyngeal lymph node metastasis has effects on DMFS and is nearly statistically significant. Teo et al. (32) suggests that distant metastases are associated with lymphatic lesion fixation and contralateral lymph node metastasis, but that the maximum diameter of lymph node is not predictive of distant metastasis. Similarly, Gao et al. (38) suggests that prognosis is neither dependent of the maximum diameter nor the area of the metastatic lymph nodes. In contrast, Lee et al. (39) reports that distant metastasis is related to the size of the lymph nodes. The results of our study show that the diameter of the metastatic lymph nodes in the neck and the inferior cervical IVa/IVb/Vb/Vc lymph node metastasis are both independent prognostic factors of DMFS for NPC patients (*P* < 0.05). In particular, we found that for each 3 cm increased in diameter of the cervical lymph nodes, DMFS decreased by more than 6%. Patients with cervical lymph nodes more than 6 cm in diameter had a 23% higher DMFS rate relative to those with 3-6 cm in diameter, indicating that a greater risk of distant metastasis for patients with larger cervical lymph nodes after IMRT. Therefore, the lymph nodes size and location are significant predictors of distant metastasis, and can be used as an important reference in clinical treatment.

The incidence of residual lymph nodes after conventional radiotherapy treatment was 33-38% (40), whereas after IMRT was about 44.7% (41) or 50% (42). In the present study, we found that 23.79% (128/538) of patients had residual cervical lymph nodes at the end of radiotherapy. Notably, the residual lymph nodes were found at the sites of the original metastatic lymph nodes,but not found in other parts. 82.8% (106/128) of the patients with residual lymph nodes were located in region II, which is consistent with the findings of Xia liangping (43). In order to examine the relationship between residual lymph nodes and prognosis, Wang Maoxin (44) performed Cox regression analysis and found that the size of residual or recurrent lymph nodes, involvement of the V region, and the number of involved areas are associated with prognosis. Liao Yulu (45) suggests that lymph node residue after radiotherapy is related to DMFS. In their study, 72% of patients achieved complete response of residual lymph nodes in the neck within 3 months of treatment, while patients with residual lymph nodes after 3 months of radiotherapy had a poor prognosis. Consistent with these findings, in our study, 74.59% (91/122) of patients had complete response of residual cervical lymph nodes within three months. Univariate and multivariate factor analyses showed that the number of lymphatic nodes, residue in unilateral or in bilateral sites, and the regions where the residues located were not risk factors of DMFS (P > 0.05). On the other hand, the size of residual lymphatic nodes and the time to residual lymphatic nodes complete response were independent prognostic factors for DMFS (P < 0.05). The DMFS rate in patients with residual lymph nodes 3 months after treatment was significantly lower than that in patients without residual lymph nodes. Therefore, the persistence of residual lymph nodes three months after radiotherapy is an independent risk factor for poor DMFS.

There are many reasons for recurrence of NPC after radiotherapy, with different presentations between local recurrence and regional recurrence. The main causes of recurrence after 2D-RT treatment include cancer biological properties (such as insensitivity to radiation of clonogenic cells), clinical stage (such as advanced T stage), and the treatment technique used. Compared with conventional radiotherapy, IMRT has been shown to improve dose distribution of locally advanced NPC patients and reduce the local recurrence caused by inadequate dose. Our study is consistent with these findings. Local recurrence after conventional radiotherapy usually occurs in the marginal low-dose areas. However, we found that local recurrence after IMRT primarily occurs in in-field high-dose areas. This is in accordance with previously published results by Jia X et al. (46). Our results also indicated that in the 60 patients that experienced the recurrence, 56 exhibited in in-field high-dose GTV. Most of these 56 recurrent patients were in advanced T stage (T3/4), which suggested that local/regional recurrence of NPC patients after IMRT treatment was related to tumor resistance and radiotherapy resistance. This result indicates that for NPC patients treated with IMRT, increasing the dose of the target can’t reduce the occurrence of local/regional recurrence. Therefore, the biological properties of cancer cells should be taken into consideration to decrease the incidence of local/regional recurrence in NPC patients.

Combining radiotherapy and chemotherapy can reduce the incidence of distant metastasis and recurrence in patients with NPC after IMRT. The risk of distant metastasis after IMRT is positively correlated with N staging, cervical metastatic lymphatic node size, residual lymphatic node size, and time for complete response of the residual lymphatic nodes. The number and regression time of cervical lymphatic nodes residues are adverse prognostic factors of DMFS after IMRT. Local recurrence primarily occurs in the first three years after treatment, is more common in males, and shows no significant relationship to T stage. The site of recurrence primarily occurs in the in-field target. Thus, when using IMRT, to further increase the tumor local control rate and DMFS, more consideration should be given to the biological properties of cancer and on radiosensitizers and the establishment of individual cancer treatments.

## Data Availability Statement

The data used to support the findings of this study are available from the corresponding author upon request.

## Ethics Statement

The studies involving human participants were reviewed and approved by The study was approved by the ethics review board of The First Affiliated Hospital of Guangxi Medical University. The patients/participants provided their written informed consent to participate in this study. Written informed consent was obtained from the individual(s) for the publication of any potentially identifiable images or data included in this article

## Author Contributions

MK conceived the original idea and wrote the proposal, MK, RW and TZ designed the study, organized the data collection, analyzed the data. MK, SC, DY, XL, YL, BY, YB, MX, PZ, ZY and KL organized the data collection and analyzed the data. MK, SC and DY wrote the manuscript for publication. DY made statistical analysis of the data, and revised the manuscript. All authors contributed to editing the manuscript and provided critical feedback and approved the final manuscript.

## Funding

This work was supported by grants from the National Natural Science Foundation of China (81460460,81760542), The Research Foundation of the Science and Technology Department of Guangxi Province, China (grants 2016GXNSFAA380252, 2018AB61001), the Research Foundation of the Health Department of Guangxi Province, China (S2018087), Guangxi Medical University Training Program for Distinguished Young Scholars (2017), Medical Excellence Award Funded by the Creative Research Development Grant from the First Affiliated Hospital of Guangxi Medical University(2016), Guangxi Medical High-level Talents Training Program, Innovation team of the First Affiliated Hospital of Guangxi Medical University.

## Conflict of Interest

The authors declare that the research was conducted in the absence of any commercial or financial relationships that could be construed as a potential conflict of interest.

## Publisher’s Note

All claims expressed in this article are solely those of the authors and do not necessarily represent those of their affiliated organizations, or those of the publisher, the editors and the reviewers. Any product that may be evaluated in this article, or claim that may be made by its manufacturer, is not guaranteed or endorsed by the publisher.

## Abbreviations

2D-RT, two-dimensional radiotherapy; ACT, adjuvant chemotherapy; CCRT, concurrent chemoradiotherapy; CR, complete response; CTV1, high-risk clinical target volume; CTV2, low-risk clinical target volume and lymphatic drainage region of the neck; DMFS, distant metastasis-free survival; GTV, gross tumor volume; IMRT, intensity-modulated radiotherapy; LRFS, local relapse-free survival; MRI, magnetic resonance imaging; NACT, neoadjuvant chemotherapy; NPC, nasopharyngeal carcinoma; OAR, organ at risk; OS, overall survival; PET/CT, positron emission tomography/computed tomography; PTV, planning target volumes; RRFS, regional recurrence-free survival; RTOG, the Radiation Therapy Oncology Group; Vr, Tumor volume with recurrence; Dmin, Minimum radiation dose for patients(min dose); Dmean, mean dose; Dmax, max dose; D95, 95% of the PTV volume received at the minimum dose; V95, 95% of the prescribed dose in the IMRT program wraps the volume of PTV; PGTVnd, Planning target volume of cervical node; PGTVnx, Planning target volume of nasopharyngeal carcinoma
